# Focus on acetabular fractures

**DOI:** 10.1007/s00068-021-01766-1

**Published:** 2021-09-27

**Authors:** Pol M. Rommens, Johannes D. Bastian

**Affiliations:** 1 Department of Orthopaedics and Traumatology, University Medical Center of Johannes Gutenberg-University Mainz, Mainz, Germany; 2 Department of Orthopaedic Surgery and Traumatology, Inselspital, Bern University Hospital, University of Bern, Bern, Switzerland

Displaced acetabular fractures have always been a major challenge for trauma surgeons. Conservative management includes immobilization in bed during several weeks and regularly results in a moderate functional outcome. For surgical treatment various surgical approaches are established [1]. A skilled selection of the appropriate surgical approach is essential as large incisions used to reach the innominate bone and acetabulum can cause severe surgical complications. Open reduction and internal fixation (ORIF) is the method of choice in patients below the age of 60. After adequate surgery, conversion to a total hip arthroplasty following posttraumatic osteoarthritis can be avoided in up to 80% of patients [2]. In patients above the age of 60, there is an ongoing discussion on the best method of treatment. ORIF is only accepted, when the joint is good reconstructible and in absence of preexisting osteoarthritis [3, 4]. In other cases, acute total hip arthroplasty is the method of choice with or without ORIF (the “Combined Hip Procedure”).

There is an ongoing research on how to predict outcome of acetabular fracture surgery. Preoperative planning through analysis of CT data, 3D printing and the development of patient-specific implants are new developments, which may help the trauma surgeon in performing a successful and less invasive surgery.

In this focus on, different authors highlight problem-oriented solutions for safe surgery of acetabular fractures. In a retrospective comparative study, Ansari et al. [5] investigated the role of preoperative 3D printing of the fractured innominate bone for the performance of the surgeon. There was a significant decrease in the operative time, amount of intraoperative blood loss and number of intraoperative fluoroscopy images in the 3D printing group. This investigation underlines the importance of 3D imaging for better understanding of the acetabular fracture anatomy. Tomaževič et al. [6] performed a comparative experimental study with 3D printed patient-specific implants and drill guides for reduction and fixation of an anterior column and posterior hemitransverse fracture. In fracture models, which were stabilized with implant-specific implants, the quality of reduction was significantly better. Patient-specific implants may facilitate retaining an accurate reduction and fixation of displaced acetabular fractures. Osterhoff et al. [7] compared the biomechanical behavior of antegrade and retrograde screw fixation of the anterior column in acetabular fractures. They did not find significant differences in construct survival, mode of failure and load to failure. These are interesting results for the surgeon, as she/he can choose the one or other screw direction depending on the surgical exposure. Fairhurst et al. [8] correlated the Estimation of Physiologic Ability and Surgical Stress (E-PASS) score with postoperative complications in acetabular fracture management. They found a significant correlation between complication rates and either the preoperative risk score (PRS) or the comprehensive risk score (CRS). They conclude that there is an important role for perioperative care in prevention, early recognition and treatment of perioperative complications. This is especially true for the older patient with an acetabular fracture and should motivate to setup an orthogeriatrie care. Bastian et al. [9] looked at the risk of intra-articular screw placement in patients with increased acetabular depth in 112 patients. There was a virtual possibility to place an infra-acetabular screw in all patients. Nevertheless, an increasing depth of the acetabulum correlated with a decrease in distance between the femoral head and the screw. Ruling out of intra-articular screw placement remains very important in every surgery.

This focus on presents original clinical and experimental research, which is connected with the management of acetabular fractures. The reader discovers the value of 3D printing for better preoperative planning and of thorough preoperative estimation of the perioperative risks. Moreover, she/he learns about the stability of antegrade and retrograde fixation of the anterior column and of the risk of joint penetration in infra-acetabular screw placement. These new aspects may help the acetabular trauma surgeon for optimal preoperative planning and performing an uneventful and high-quality surgery. We wish you very interesting readings.

Prof. Dr. Dr. h. c. Pol M. Rommens

Guest-editor

Prof. Dr. Johannes D. Bastian

Guest-editor
